# Sulphonamide inhibition studies of the β-carbonic anhydrase from the bacterial pathogen *Clostridium perfringens*

**DOI:** 10.1080/14756366.2017.1388233

**Published:** 2017-11-03

**Authors:** Daniela Vullo, R. Siva Sai Kumar, Andrea Scozzafava, James G. Ferry, Claudiu T. Supuran

**Affiliations:** a Chemistry Department, Laboratorio di Chimica Bioinorganica, Università degli Studi di Firenze, Florence, Italy;; b Department of Biochemistry and Molecular Biology, Eberly College of Science, The Pennsylvania State University, University Park, PA, USA;; c NEUROFARBA Department, Sezione di Scienze Farmaceutiche e Nutraceutiche, Università degli Studi di Firenze, Florence, Italy

**Keywords:** Carbonic anhydrase, sulphonamide, sulphamate, dorzolamide, clostridium perfringens, β-class enzyme

## Abstract

The β-carbonic anhydrases (CAs, EC 4.2.1.1) from the pathogenic bacterium *Clostridium perfringens* (CpeCA) was recently characterised kinetically and for its anion inhibition profile. In the search of effective CpeCA inhibitors, possibly useful to inhibit the growth/pathogenicity of this bacterium, we report here an inhibition study of this enzyme with a panel of aromatic, heterocyclic and sugar sulphonamides/sulphamates. Some sulphonamides, such as acetazolamide, ethoxzolamide, dichlorophenamide, dorzolamide, sulthiame and 4-(2-hydroxymethyl-4-nitrophenyl-sulphonamido)ethylbenzenesulphonamide were effective CpeCA inhibitors, with K_I_s in the range of 37.4–71.6 nM. Zonisamide and saccharin were the least effective such inhibitors, whereas many other aromatic and heterocyclic sulphonamides were moderate – weak inhibitors with K_I_s ranging between 113 and 8755 nM. Thus, this study provides the basis for developing better clostridial enzyme inhibitors with potential as antiinfectives with a new mechanism of action.

## 1. Introduction

Carbonic anhydrases (CAs, EC 4.2.1.1) are metalloenzymes present in all three of life’s phylogenetic domains (*Bacteria*, *Archaea* and *Eukarya*) and various isoforms present in most organisms investigated so far^1–12^. By converting metabolism-generated CO_2_ to soluble products, bicarbonate and protons, these enzymes are crucial in a multitude of physiologic processes connected among others with pH homoeostasis, biosynthetic reactions in which CO_2_/bicarbonate are involved, electrolyte secretion, photosynthesis, etc.^1–12^. The α-class CAs present in vertebrates, including humans^1–6^, are drug targets for obtaining antiglaucoma agents^13^^,^^14^, anticonvulsants^15^, drugs for the treatment of idiopathic intracranial hypertension and other neurologic disorders^15^^,^^16^, antiobesity agents^17^ and diuretics^18–20^. Most of these clinically used CA inhibitors (CAIs) belong to the sulphonamide class, as they possess the primary sulphonamide (or its isosteres, sulphamate and sulphamide moieties) as the zinc-binding function^1–6^^,^^21^. Indeed, these compounds bind (in deprotonated, anionic form) to the CA active-site zinc ion, which is crucial for the catalytic activity^21^. Representatives of this class of pharmacological agents have multiple therapeutic applications for decades, although many of these first-/second-generation agents do show side effects connected with the inhibition of off-target isoforms, due to the fact that in humans there are 15 CAs which do not differ significantly in their active site architecture^3–5^^,^^22^ and most of them show high affinity for this class of CAI^1–6^. However, in the last decade, a large number of different classes of CAIs and diverse inhibition mechanisms were reported, with a range of new chemotypes such as the coumarins^23–26^, sulphocoumarins^27^^,^^28^, polyamines^29^, dithiocarbamates^30^^,^^31^ and carboxylates^32^^,^^33^ among others. Many of these novel types of CAIs show significant isoform-selective inhibition profiles, making this class of drugs much more attractive as candidates for the development of new generation pharmacological agents^4^^,^^23–33^.

Bacteria encode CAs belonging to three classes, the α-, β- and γ-CAs^7^^,^^34–40^. These enzymes seem to be involved in crucial metabolisms, which probably explains both their wide distribution in Gram-negative and Gram-positive bacteria, as well as their generally very effective catalytic properties for the hydration of CO_2_ to bicarbonate and protons^34–42^. Thus, ultimately, inhibition of bacterial CAs has been proposed as an alternative approach for obtaining antibiotics with an alternative mechanism of action compared to the classical drugs that interfere with bacterial cell wall biosynthesis, DNA-gyrase or similar such targets, which led to an extensive drug resistance phenomenon^7^^,^^12^^,^^34^^,^^36^.

In previous work from our groups, we have reported the cloning and characterisation of a new β-CA from the bacterial pathogen *Clostridium perfringens*, CpeCA [41] that has also been investigated for its interaction with anions and other small molecules known to interact with metalloenzymes such as CA^42^. We previously observed that most anions are millimolar CpeCA inhibitors, whereas sulphamate, sulphamide, phenylboronic acid and phenylarsonic acid are the most effective inhibitors, with K_I_s in the range of 7–75 μM. Thus, no highly effective CpeCA inhibitors were detected so far and this is the reason why we investigated the interaction of this enzyme with sulphonamides and sulphamates, the class of CAIs which usually leads to effective antimicrobial agents.

## 2. Materials and methods

### 2.1. Chemistry

Compounds **1**–**24** and **AAZ**-**HCT** were commercially, highest purity available derivatives from Sigma-Aldrich (Milan, Italy) and were used without further purification or were prepared as reported earlier by our group^43–51^.

### 2.2. Carbonic anhydrase assay

An applied photophysics stopped-flow instrument has been used for assaying the CA catalysed CO_2_ hydration activity [52]. Phenol red (at a concentration of 0.2 mM) has been used as indicator, working at the absorbance maximum of 557 nM, with 20 mM TRIS (pH 8.3) as buffer, and 20 mM Na_2_SO_4_ (for maintaining constant the ionic strength), following the initial rates of the CA-catalysed CO_2_ hydration reaction for a period of 10–100 s. The CO_2_ concentrations ranged from 1.7 to 17 mM for the determination of the kinetic parameters and inhibition constants. For each inhibitor, at least six traces of the initial 5–10% of the reaction have been used for determining the initial velocity. The uncatalysed rates were determined in the same manner and subtracted from the total observed rates. Stock solutions of inhibitor (0.1 mM) were prepared in distilled-deionised water and dilutions up to 0.01 nM were done thereafter with the assay buffer. Inhibitor and enzyme solutions were pre-incubated together for 15 min at room temperature prior to assay, in order to allow for the formation of the E–I complex. The inhibition constants were obtained by non-linear least-squares methods using PRISM 3 and the Cheng–Prusoff equation, as reported earlier^23–27^ and represent the mean from at least three different determinations. All CA isofoms were recombinant ones obtained in-house as reported earlier^41^^,^^42^.

## 3. Results and discussion

We investigated the inhibition of CpeCA with a panel of sulphonamides of type **1–24**, which include both aromatic and heterocyclic derivatives, employed extensively for the design of various classes of CAIs with interesting physicochemical properties^43–51^ (Figure 1). The clinically used agents acetazolamide **AAZ**, methazolamide **MZA**, ethoxzolamide **EZA**, dichorophenamide **DCP**, dorzolamide **DZA**, brinzolamide **BRZ**, benzolamide **BZA**, topiramate **TPM**, zonisamide **ZNS**, sulpiride **SLP**, indisulam **IND**, valdecoxib and **VLX**, celecoxib **CLX**, sulthiame **SLT**, saccharin **SAC** and hydrochlorothiazide **HCT** [9] were also included in this study as they incorporate the sulphonamide/sulphamate zinc-binding function and act as potent CAIs against many β-CAs investigated earlier [7]. The inhibition observed with these derivatives against CpeCA and the human (h) off-target α-class enzymes hCA I and II, are shown in Table 1.

Figure 1.Structure of sulphonamides investigated as CAIs in this work.

**Figure:**
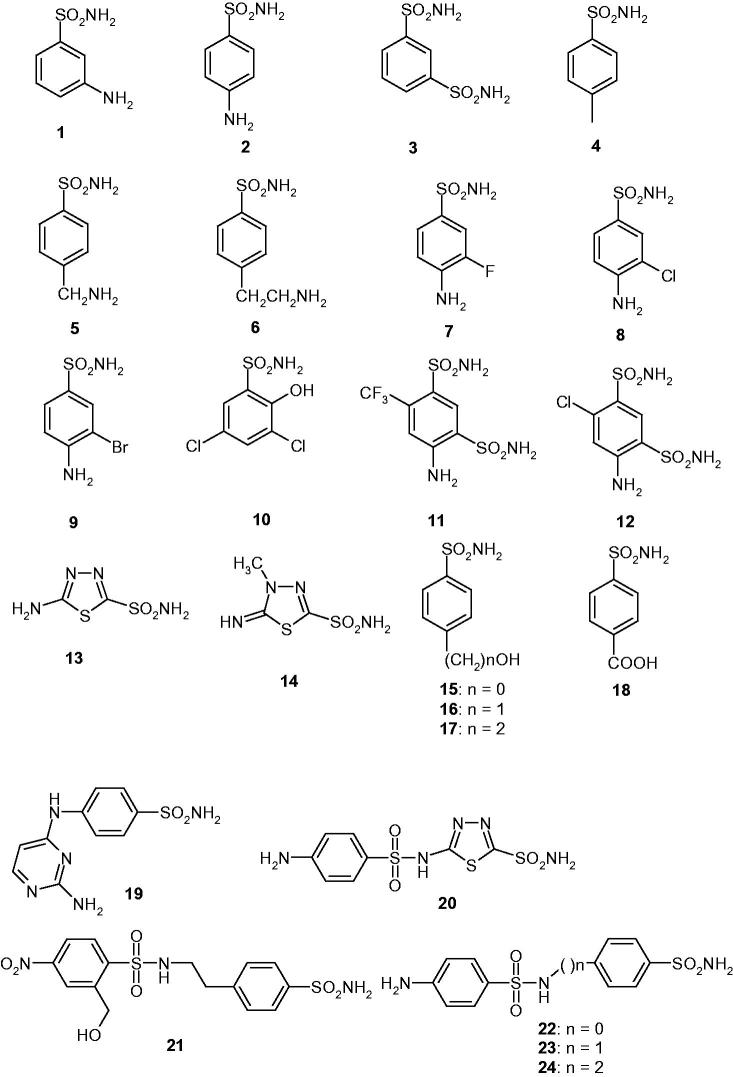

**Figure:**
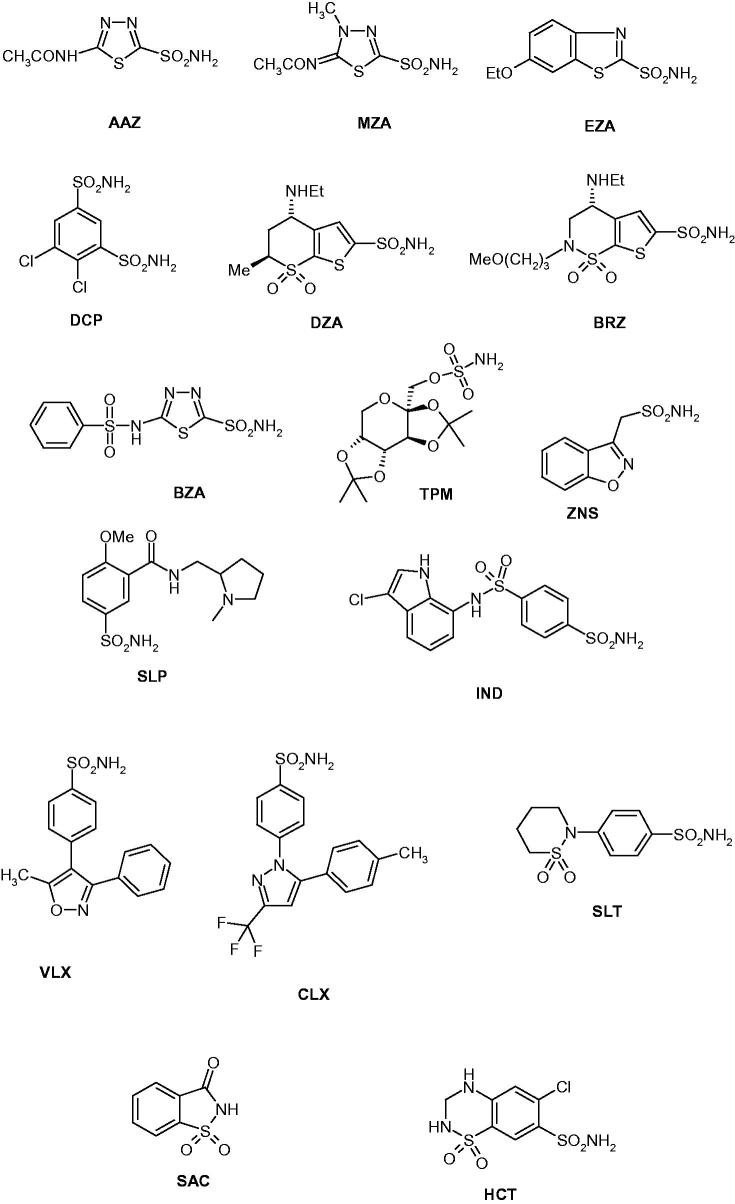

**Table 1.** Inhibition of human isoforms hCA I and hCA II (off-target enzymes), as well as the bacterial enzyme from *C. perfringens* (CpeCA) with sulphonamides **1–24** and the clinically used drugs **AAZ–HCT**, by a stopped-flow, CO_2_ hydrase assay [52].

	*K*_I_* (nM)		
Inhibitor/Enzyme class	hCA I^a^α	hCA II^a^α	CpeCA^b^β
**1**	28,000	300	451
**2**	25,000	240	402
**3**	79	8	210
**4**	78,500	320	379
**5**	25,000	170	3690
**6**	21,000	160	2430
**7**	8300	60	443
**8**	9800	110	320
**9**	6500	40	713
**10**	7300	54	4670
**11**	5800	63	8755
**12**	8400	75	7635
**13**	8600	60	297
**14**	9300	19	460
**15**	5500	80	131
**16**	9500	94	145
**17**	21,000	125	314
**18**	164	46	1268
**19**	109	33	179
**20**	6	2	105
**21**	69	11	697
**22**	164	46	513
**23**	109	33	312
**24**	95	30	51.2
**AAZ**	250	12	49.1
**MZA**	50	14	113
**EZA**	25	8	71.6
**DCP**	1200	38	68.0
**DZA**	50,000	9	37.4
**BRZ**	45,000	3	121
**BZA**	15	9	497
**TPM**	250	10	204
**ZNS**	56	35	>100,000
**SLP**	1200	40	389
**IND**	31	15	173
**VLX**	54,000	43	160
**CLX**	50,000	21	312
**SLT**	374	9	63.1
**SAC**	18,540	5959	>100,000
**HCT**	328	290	219

* Errors in the range of 5–10% of the shown data, from three different assays.

a Human recombinant isozymes, stopped flow CO_2_ hydrase assay method, from reference [3–5].

b Recombinant bacterial enzyme, stopped flow CO_2_ hydrase assay method, this work.

The following structure–activity relationship (SAR) can be drawn from the data of Table 1 regarding CpeCA inhibition with these compounds

- The least effective CpeCA inhibitors were zonisamide and saccharin, which did not affect the enzyme activity up to 100 µM (Table 1). **ZNS** is in fact the only aliphatic sulphonamide, whereas **SAC** the only secondary, acylated sulphonamide among the investigated compounds.
- Moderate–weak inhibitory action, in the micromolar range, was observed for the following sulphonamides: **5, 6, 10–12** and **18**, which had K_I_s in the range of 1.268–8.755 µM. These compounds belong to the aminoalkyl–benzenesulphonamide (**5** and **6**) and tetrasubstituted benzenesulphonamide/disulphonamide (**10**–**12**) series. Probably, the large number of substituents on the phenyl ring for the last type of derivatives is detrimental to their efficient binding to the enzyme.
- More effective but moderate CpeCA inhibitors were the following compounds: **1**–**4, 7**–**9, 13, 14, 17, 19, 21**–**23, BZA, TPM, SLP**–**CLX** and **HCT**, which had K_I_s in the range of 160–713 nM. It is obvious that these derivatives belong to a variety of different classes, with both aromatic, heterocyclic and sugar derivatives among them. Thus, a real SAR is difficult to draw, but it is important to note that many structural variations in the scaffold of aromatic/heterocyclic sulphonamides are tolerated without a significant loss of the CpeCA inhibitory action.
- The most effective CpeCA inhibitors were **15, 16, 20, 24, AAZ, MZA, EZA, DCP, DZA, BRZ** and **SLT**, which showed K_I_s in the range of 37.4–145 nM (Table 1). Again many different chemotypes led to quite effective CAIs, among which the most notable are dorzolamide, a rather bulky bicyclic sulphonamide (the best inhibitor with a *K*_I_ of 37.4 nM), acetazolamide (the second best inhibitor with a *K*_I_ of 49.1 nM) as well as the aromatic compound 4–(2-hydroxymethyl-4-nitrophenyl-sulphonamido)ethylbenzenesulphonamide **24**, with a *K*_I_ of 51.2 nM (Table 1). All of them are highly different structurally, which is of extreme importance for the possible design of even better CpeCA inhibitors belonging to the sulphonamide class.
- The off-target isoforms hCA I and II have a very different inhibition profile with the compounds investigated here (Table 1), whereas hCA I has generally a lower affinity for most of these inhibitors, hCA II is highly inhibited by most of them, usually in the low nanomolar range, which makes it quite difficult to obtain CpeCA-selective inhibitors form this class of agents.

## 4. Conclusions

Species belonging to the genus Clostridium, such as *Clostridium tetani*, *C. botulinum*, *C. barati*, *C. butirycum*, *C. hystolyticum* and *C. perfringens* among others, are strictly anaerobic pathogens that provoke serious human disease, such as tetanus, botulism, gas gangrene, bacterial corneal keratitis and other infections^53^^,^^54^. Although some progress has been achieved ultimately for designing pharmacological agents effective against these diseases, such as for example protease inhibitors targeting various metalloproteases essential for the life cycle of these pathogens, there is a constant search for novel drug targets that may lead to new classes of such agents, considering the serious antibiotic drug resistance problems emerging worldwide with the clinically used drugs^53^^,^^54^. In the search of effective compounds interfering with the metabolism of these pathogens, in this paper, we investigated potential CpeCA inhibitors, possibly useful to inhibit the growth/pathogenicity of this bacterium. A panel of aromatic, heterocyclic and sugar sulphonamides/sulphamates were employed for the inhibition of this bacterial β-class enzyme. Some sulphonamides, such as acetazolamide, ethoxzolamide, dichlorophenamide, dorzolamide, sulthiame and 4-(2-hydroxymethyl-4-nitrophenyl-sulphonamido)ethylbenzenesulphonamide were effective CpeCA inhibitors, with K_I_s in the range of 37.4–71.6 nM. Zonisamide and saccharin were the least effective inhibitors, whereas many other aromatic and heterocyclic sulphonamides were moderate–weak inhibitors with K_I_s ranging between 113 and 8755 nM. This study thus provides the basis for developing better clostridial enzyme inhibitors with potential as antiinfectives with a new mechanism of action.
